# Antibody response against koala retrovirus (KoRV) in koalas harboring KoRV-A in the presence or absence of KoRV-B

**DOI:** 10.1038/s41598-019-48880-0

**Published:** 2019-08-27

**Authors:** O. Olagoke, B. L. Quigley, M. V. Eiden, P. Timms

**Affiliations:** 1 Genecology Research Center, University of the Sunshine Coast, 90 Sippy Downs Drive, Sippy Downs, 4556 Queensland, Australia; 2 Section on Directed Gene Transfer, Laboratory of Cellular and Molecular Regulation, National Institute of Mental Health, National Institutes of Health, Bethesda, Maryland USA

**Keywords:** Humoral immunity, Retrovirus, Viral host response

## Abstract

Koala retrovirus (KoRV) is in the process of endogenization into the koala (*Phascolarctos cinereus*) genome and is currently spreading through the Australian koala population. Understanding how the koala’s immune system responds to KoRV infection is critical for developing an efficacious vaccine to protect koalas. To this end, we analyzed the antibody response of 235 wild koalas, sampled longitudinally over a four-year period, that harbored KoRV-A, and with or without KoRV-B. We found that the majority of the sampled koalas were able to make anti-KoRV antibodies, and that there was a linear increase in anti-KoRV IgG levels in koalas up to approximately seven years of age and then a gradual decrease thereafter. Koalas infected with both KoRV-A and KoRV-B were found to have slightly higher anti-KoRV IgG titers than koalas with KoRV-A alone and there was an inverse relationship between anti-KoRV IgG levels and circulating KoRV viral load. Finally, we identified distinct epitopes on the KoRV envelope protein that were recognized by antibodies. Together, these findings provide insight into the koala’s immune response to KoRV and may be useful in the development of a therapeutic KoRV vaccine.

## Introduction

Koala retrovirus (KoRV) has been reported to play a key role in neoplasia and chlamydial diseases of both captive and wild koala populations^1–4^. A seven year old survey to determine the prevalence of KoRV in Australia previously reported that all koalas tested from northern Australia (New South Wales and Queensland) contain detectable KoRV provirus, while 15–72% of koalas from southern Australia (Victoria and South Australia) also have detectable KoRV provirus^5^. A total of nine envelope protein variants of KoRV (KoRV-A to KoRV-I) have been identified^6^, two of which have been widely studied (KoRV-A and KoRV-B). KoRV-A has been shown to use a phosphate transporter (PiT1)^7^ for cell attachment and entry, while KoRV-B uses the thiamine transporter 1 (THTR1)^1^. The patterns of KoRV integration in the koala genome suggest that KoRV is currently undergoing endogenization into the koala^8,9^, offering a unique opportunity to study the endogenization process of a retrovirus in real time, as well as the host’s immune response to this invasion.

Northern koalas appear to harbor endogenous KoRV-A based on conserved genome integration pattern determined by southern blot analysis and relatively high provirus copy number per cell (~165 copies per cell)^5,8,9^. KoRV-A integrants in northern koalas display a pattern consistent with that of an endogenous retrovirus (with some loci integrated in the same genomic location in all cells) and of an infectious retrovirus (with other loci integrated randomly in infected cells)^9^. The characterization of replication competent KoRV-A molecular clones further supports the supposition that KoRV-A is infectious in northern koalas^10,11^. Collectively, the evidence to date shows KoRV-A as a circulating subtype of KoRV with provirus transmitted both horizontally and vertically in northern koalas. This is quite different to the information available about KoRV-A in southern koalas, where low infection rates and fewer KoRV proviral copies per cell (~0.00013 copies per cell) suggest only exogenous KoRV variants (including KoRV-A) are present in these populations^5^. New information from a recent study in preprint has raised the possibility of endogenous KoRV in southern koalas, based on detecting apparently defective provirus^12^. However, until this interesting finding can be investigated more thoroughly, the current body of evidence from southern Australia only supports the presence of exogenous KoRV in this region.

The remaining KoRV variants, KoRV-B to KoRV-I, have been detected and studied in northern koala populations^6^. Contrasting with KoRV-A, there has been no evidence to suggest that any of these eight KoRV variants have endogenized in the northern koala population^1,9^. Testing of southern koala populations for non-KoRV-A variants has traditionally been limited to conventional PCR for KoRV-B provirus, which has been undetectable to date, leaving the impression that southern koalas harbor only KoRV-A^13^. However, a recent study has revealed genetic evidence of non-KoRV-A variants in southern koalas, suggesting the presence and diversity of KoRV in the south may be more complicated that currently recognized^14^.

The possible involvement of viruses, like KoRV, in the development of disease in koalas, and particularly neoplasia, was first suggested in 1961^15^. While there has been no definitive and direct measurement of koala health outcomes in response to experimental KoRV infection, observational studies in wild and captive koalas do support possible roles for KoRV in koala pathologies. For instance, having detectable levels of KoRV-A provirus in southern koalas has been associated with chlamydial disease^13^. Similarly, detectable levels of KoRV-B provirus in northern koalas has also been associated with clinical chlamydial disease and neoplasia^16,17^. Finally, significant increases in total KoRV viral load (not defined by variant) has also been found to be associated with the development of lymphoma^4^. These statistically significant associations suggest KoRV presence and expression have some connection to serious health conditions in koalas.

A common feature of retroviral infections is the ability to trigger a host immune response^18,19^. While this natural immune response is not always protective, it can confer resistance against the establishment of infection and disease in some individuals. The innate immune system plays a significant role in retrovirus control, with downregulation of nucleic acid-recognizing toll-like receptor (TLRs) 3, 7 and 9 being associated with reactivation of murine leukemia virus (MLV)^20,21^. Cell mediated immunity also contributes, with levels of virus-specific cytotoxic T lymphocytes being shown to play a critical role in determining feline leukemia virus (FeLV) infection outcomes in cats^22,23^. Adaptive immunity, such as T cell and B cell responses, can also be induced against endogenous retroelements by using experimental immunization, as seen in mouse studies using gp70 to target murine leukemia virus and non-human primate studies using Gag and Env proteins to target simian endogenous retrovirus-K^24,25^. Finally, antibodies play a key role in defending against exogenous retroviruses^26,27^. Antibody titers against gibbon ape leukemia virus and bovine leukemia virus have been shown to sometimes correlate, inversely, with viral load and disease severity^28–30^. Similarly, passive transfer of FeLV neutralizing antibodies via colostrum to kittens has been shown to provide some protection against viremia upon subsequent exposure^31^. However, antigen-specific antibody titers do not stay static throughout an infection, with age, sex and environmental factors having been shown to influence antigen-specific antibody levels in infected individuals^32,33^. Collectively, the antibody response to retrovirus infection is important for virus containment.

Aging has been associated with reduction in the number of functional innate immune cells^32^ and subsequent reduction in ability to clear new infections^34^. Similarly, production of new lymphocytes declines with aging^35,36^. Overall, there is a general decline in immune system function with age, known as immunosenescence^37^. The relationship between infection load and age appears to be complicated. Whereas an association between viral load and age has been reported for certain viruses including KoRV^4^, there appears to be no significant relationship between age and viral load in FeLV, Feline foamy virus, and HTLV^38,39^.

While there have been studies aimed at developing vaccines against KoRV^40–42^, very little is known about how the koala’s immune system responds to KoRV infection. A major concern is whether an immune response can be mounted to an endogenous KoRV-A virus. It has been proposed that the expression of endogenous retroviral proteins in embryonic tissues may trigger immune tolerance to these proteins in the developing immune system^43^. Escape from immune tolerance has been reported for some infectious polytropic viruses, despite the expression of their antigens during ontogenesis^44^. However, a study of a small group of northern koalas (n = 16) did not find antibodies against KoRV, suggesting that koalas with endogenous KoRV infection may be tolerant to KoRV^41^. Subsequently, a study of immune cytokine expression in lymphocytes obtained from northern koalas reported an association between altered immune cytokines and KoRV-B infection^45^, suggesting a role for at least some variants of KoRV in the response to KoRV infection. However, more study into the immune response to KoRV in koalas known to contain both endogenous and exogenous variants of KoRV is needed.

To this end, we tested 235 northern koalas and demonstrated that koalas harboring KoRV-A, with or without KoRV-B, do indeed produce anti-KoRV IgG antibodies. We showed an association between both koala age and the presence of KoRV-B with anti-KoRV IgG levels. We then went on to identify distinct antibody epitopes on the ectodomain of the transmembrane subunit of KoRV envelope protein. A key feature of our study was our ability to identify and monitor cohorts of koalas over time, thus enabling us to test several of our hypothesis on longitudinal samples.

## Results

### KoRV status of animals included in the study

Two hundred and thirty-five koalas from Gold Coast City Region (GCCR) and Moreton Bay Region (MBR) populations were screened for KoRV infection using KoRV-A and KoRV-B specific PCR assays, to detect integrated provirus. All 235 koalas tested positive for KoRV-A envelope gene (100% prevalence), while only 27% (n = 63/235) of koalas were positive for KoRV-B envelope gene.

### Anti-KoRV IgG response in infected koalas

**Figure 1:**
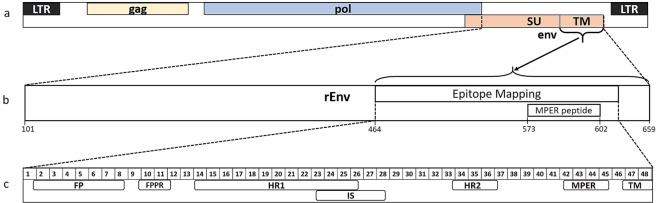
Schematics showing KoRV genome and relevant segments used in this study (modified from Olagoke *et al*.^42^). (**a**) Representative KoRV genome showing coding regions. (**b**) Segment of the env protein used to produce recombinant protein (rEnv) and synthetic peptide (MPER peptide), and for epitope mapping. The numbers shown represent corresponding amino acid number (GenBank: AF151794). (**c**) Layout of the transmembrane subunit of KoRV envelope protein showing important regions. The numbers represent 15mer amino acids peptides with three amino acids offset. FP = fusion peptide, FPPR = fusion peptide proximal region, HR1 = heptad repeat 1, IS = immunosuppressive domain, HR2 = heptad repeat 2, MPER = membrane proximal external region, TM = transmembrane region.

**Figure 2:**
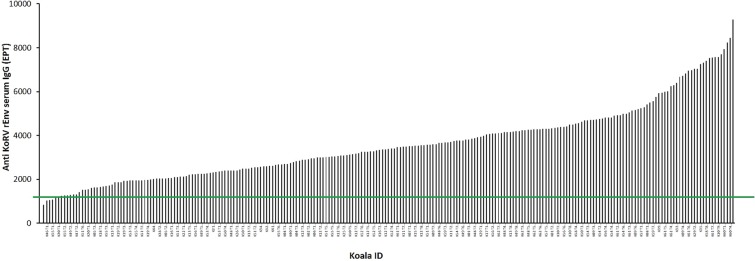
Anti-KoRV rEnv IgG levels expressed as end-point titers (EPT) in serum samples of KoRV-A positive koalas (n = 235). The green line indicates the assay background.

Using our previously described ELISA based on a recombinant KoRV-A envelope (env) protein (rEnv)^42^ (Fig. 1), we measured anti-KoRV IgG levels in koala serum (n = 235). The majority (n = 223/235, 95%) of the animals had anti-KoRV IgG antibodies in their serum, with titers ranging from just above background (end point titer (EPT) of 1000), to EPTs of 9000 (Fig. 2). When we compared the results from the two geographical areas under study, GCCR (n = 28) and MBR (n = 207) using an unpaired t-test, the two populations had similar levels of anti-KoRV IgG antibodies (t(233) = 1.065, p = 0.288) (Supplementary Fig. 1).

### Association of anti-KoRV IgG levels with age

**Figure 3:**
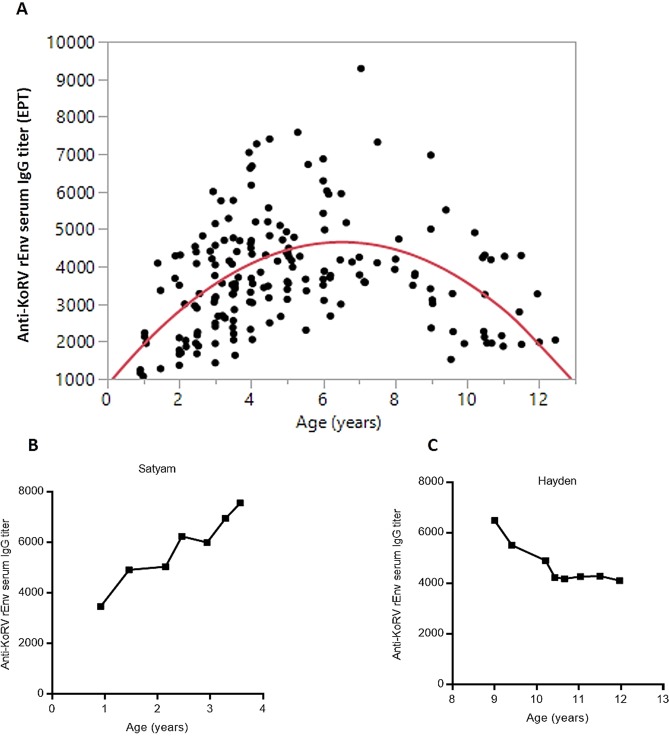
Association between anti-KoRV IgG levels expressed as end-point titers (EPT) in koala serum and age of the koala. (**A**) Association was tested using regression analysis (second order polynomial) at a population level in 207 koalas. The level of significance was measured as p < 0.001. (**B**,**C**) are examples of serum IgG measured in young and old koalas respectively over a minimum of three years. All 14 koalas are shown in Supplementary Fig. 2.

Examining the anti-KoRV IgG titers from the MBR koala population (n = 207) in the context of the koala’s age, we found that there was a gradual increase in anti-KoRV IgG levels between ages one and six, peaking at around seven to eight years and then declining in koalas aged nine 12 (Fig. 3). To better understand the relationship between age and anti-KoRV IgG levels, we analyzed serum samples from a subset of koalas (n = 14) that were sampled periodically over three to four years. Consistent with our population data, we found that the anti-KoRV IgG levels in individual koalas increased from one to around five years of age. Again, titers declined in older aged animals (Fig. 3, Supplementary Fig. 2).

### Association between KoRV-B infection and anti-KoRV IgG levels

**Figure 4:**
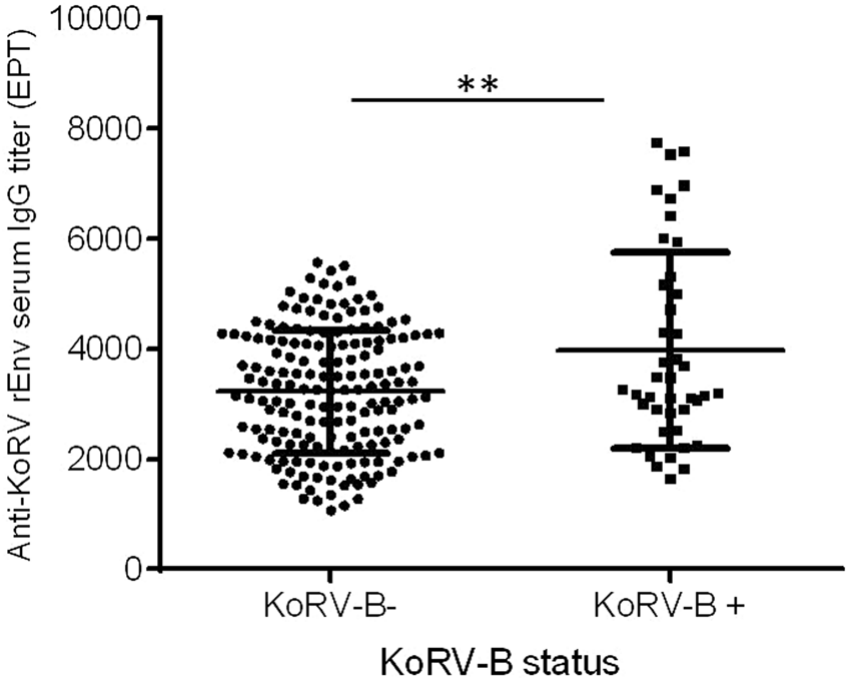
Anti-KoRV rEnv IgG levels expressed as end-point titers (EPT) in serum samples from KoRV-B negative (n = 186) and KoRV-B positive (n = 42) koalas compared and presented as mean ± SD. The level of significance was measured using student’s T-test (p < 0.05).

To determine if the presence of KoRV-B in koalas carrying KoRV-A has any association with anti-KoRV IgG antibody levels, we compared sera from KoRV-B positive and negative koalas (Fig. 4). Using unpaired student’s t test, sera from koalas with KoRV-B infection were found to have higher levels of anti-KoRV antibodies compared to KoRV-B negative sera (t (226) = 3.424, p = 0.0007).

### Association between KoRV RNA expression load and anti-KoRV IgG levels

**Figure 5:**
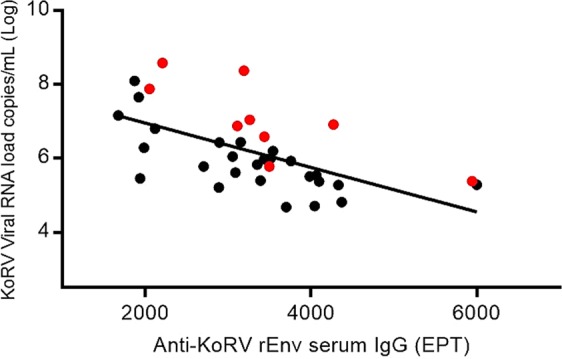
Relationship between plasma KoRV viral RNA load (copies/mL) as measured by qPCR in plasma of koalas and serum anti-KoRV IgG levels (EPT) in paired plasma and serum samples. KoRV-B negative koalas (n = 27) are shown in black circles while KoRV-B positive koalas (n = 9) are shown in red circles (R^2^ = 0.53, p = 0.011, n = 34).

To investigate the relationship between circulating viral load and anti-KoRV IgG levels, we quantified total KoRV plasma viral RNA using the *pol* gene and correlated it with serum anti-KoRV IgG levels in paired samples from 34 randomly chosen koalas. These koalas were either positive for KoRV-A and KoRV-B, or positive for KoRV-A only. The results showed a negative correlation between plasma KoRV viral RNA load and anti-KoRV IgG levels in koalas that were positive for KoRV-A only (R^2^ = 0.54, p ≤ 0.001, n = 27) and for koalas positive for both KoRV-A and KoRV-B (R^2^ = 0.61, p = 0.013, n = 9). Overall, expressed KoRV viral load declined as anti-KoRV IgG increased (R^2^ = 0.53, p = 0.011, n = 34) (Fig. 5). We also quantified and compared plasma KoRV viral RNA load with serum anti-KoRV IgG levels in 14 koalas (ages 0.92 to 12.5 years) sampled over a period of three to four years. Overall, the data obtained from these koalas over time did not yield any specific pattern; rather each koala appeared to have its unique pattern of expressed virus load and anti-KoRV IgG antibody level (Supplementary Fig. 3).

### Characterization of epitopes within the ectodomain of KoRV transmembrane protein recognized following natural infection

**Figure 6:**
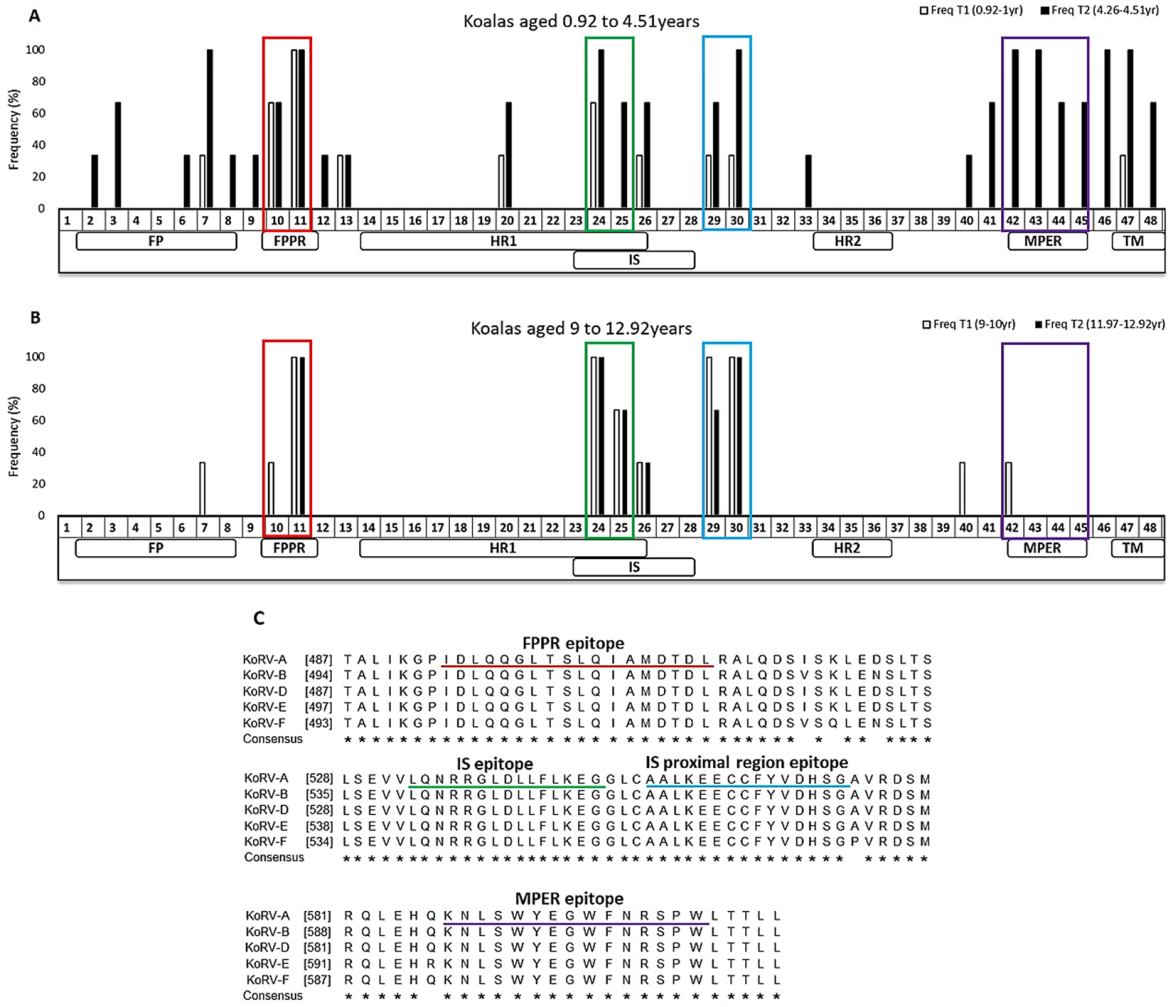
p15E ectodomain B cell epitope mapping in two groups of KoRV-A positive koalas sampled over two time points: (**A**) dependent juveniles (n = 3) (0.9–1 year, white bars) and again as adults (4.5 years, black bars), and (**B**) older koalas (n = 3) (9–10 years, white bars) and again at 12–12.9 years (black bars). The numbers on the x-axis represent 15-mer amino acids peptides with three amino acids offsets spanning the ectodomain of KoRV-A env protein and the boxes represent epitopes of interest. (**C**) Comparison of amino acid sequences around distinct epitopes (colored boxes and lines) on KoRV p15E ectodomain among different KoRV subtypes. Conserved amino acid residues are indicated by *. FP = fusion peptide, FPPR = fusion peptide proximal region, HR1 = heptad repeat 1, IS = immunosuppressive domain, HR2 = heptad repeat 2, MPER = membrane proximal external region, TM = transmembrane region.

To identify which epitopes on the ectodomain of the transmembrane subunit (p15E) of KoRV env protein were recognised in koalas infected with KoRV-A, we designed 48 overlapping 15mer peptides with three amino acids offsets, spanning the ectodomain of the p15E subunit of KoRV-A env protein (Fig. 1). Peptides 47 and 48 contained the initial three and six amino acids, respectively, in the endodomain region of p15E. To identify which epitopes were recognised over the koala’s lifespan, we selected six KoRV-B negative koalas from two distinct age groups (0.92 to 4.5 years and 9 to 12.9 years) with three animals per group. Each koala was assayed at the first time of sampling and again approximately three years later. We observed that dependent juveniles (about a year old) recognized 10 peptides across the region assessed (33–100% frequency; peptides 7, 10, 11, 13, 20, 24, 26, 29, 30, 47) (Fig. 6A). When sera obtained three years later from the same animals were assessed, 16 additional peptides were recognized, bringing the total to 26 peptides (33–100% frequency; peptides 2, 3, 6, 8, 9, 12, 25, 33, 40–46, 48) (Fig. 6A). In the second group, older koalas between the ages of nine and 10 recognized 10 peptides (33–100% frequency; peptides 7, 10, 11, 24–26, 29, 30, 40, 42) (Fig. 6B). After three years, only six of these peptides were still recognized (33–100% frequency; peptides 11, 24–26, 29, 30) (Fig. 6B). Taken together, we observed a pattern in which a small number of epitopes were recognized early in life, increasing to more epitopes in adult koalas, and finally declining in number in older koalas.

Three distinct epitopes recognized across all age groups were identified in this study. Peptides 10 and 11, located on the fusion peptide proximal region of the p15E protein, were recognized by antibodies from 67% of dependent juveniles and 100% of adult koalas (Fig. 6, red boxes and line). Peptides 24 and 25, located within the immunosuppressive domain of the p15E protein, were recognised by antibodies from at least 67% of adult koalas while peptide 24 was recognised by antibodies from 67% of dependent juvenile koalas (Fig. 6, green boxes and line). Peptides 29 and 30, located within the immunosuppressive domain proximal region, were recognized at 67% and 100% frequency by antibodies from adult koalas and 33% of dependent juveniles (Fig. 6, blue boxes and line). A fourth epitope, peptides 42–45, spanning the membrane proximal external region (MPER), was recognised by antibodies from adult koalas (67–100%) (Fig. 6, purple boxes and line), while there was minimal to no recognition in aged koalas and dependent juveniles respectively.

### Some epitopes recognized in animals with KoRV-A are also recognised in animals vaccinated with recombinant KoRV env protein

**Figure 7:**
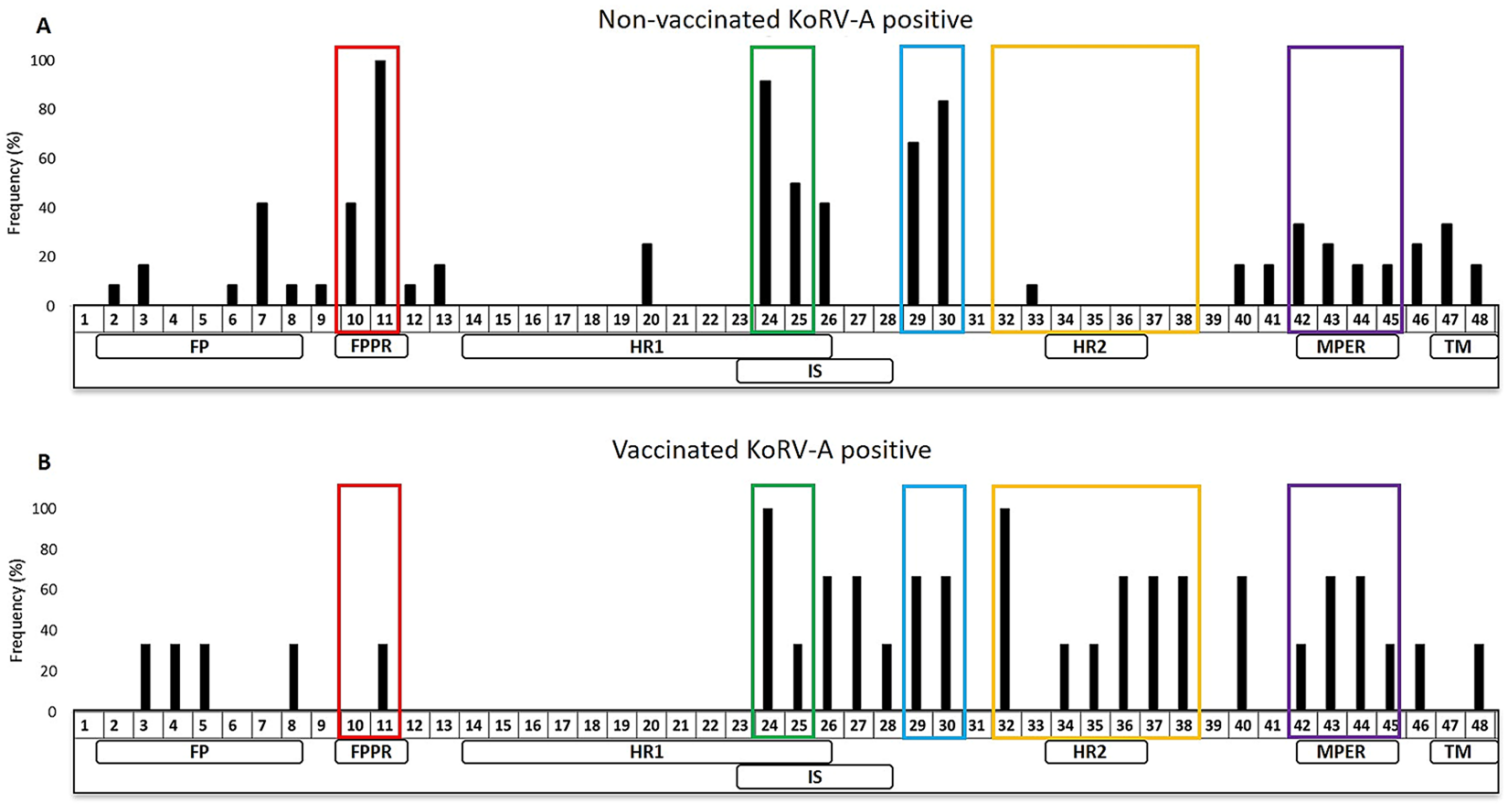
Comparison of p15E ectodomain B cell epitopes recognized in (**A**) non-vaccinated northern koalas harbouring KoRV-A (n = 12) and (**B**) vaccinated southern koalas believed to be infected with exogenous KoRV-A (n = 3). The numbers on the x-axis represent 15-mer amino acids peptides with three amino acids offsets spanning the ectodomain of KoRV-A env protein and the colored boxes represent regions of interest. FP = fusion peptide, FPPR = fusion peptide proximal region, HR1 = heptad repeat 1, IS = immunosuppressive domain, HR2 = heptad repeat 2, MPER = membrane proximal external region, TM = transmembrane region.

We recently reported the development of a tri-adjuvanted recombinant KoRV envelope protein based vaccine in koalas infected with KoRV-A from southern Australia^42^. To compare how anti-KoRV antibodies from these southern koalas compared to northern koalas (where KoRV-A is present in both endogenous and infectious forms), we compared all epitopes recognized by antibodies from six northern koalas in the present study, sampled twice, approximately three years apart (n = 12), with the vaccinated southern koalas (n = 3). For the vaccinated koalas, the response of each pre-vaccination sample was compared against its 8 weeks post-vaccination sample. The results presented for the non-vaccinated koalas represent an average of the frequency of each reported epitope from Fig. 6. Our results showed that peptides spanning the immunosuppressive domain (peptides 24–26) and the MPER region (peptides 42–45) that were recognized by non-vaccinated KoRV-A positive northern koalas were also recognised by the vaccinated KoRV-A positive southern koalas (Fig. 7). In addition to these peptides, the vaccinated southern koalas also recognized peptides around the heptad repeat 2 domain (peptides 32, 34–38) (Fig. 7). Interestingly, these peptides were not recognised by the non-vaccinated northern koalas.

### Antibodies against MPER correlates with rEnv IgG

**Figure 8:**
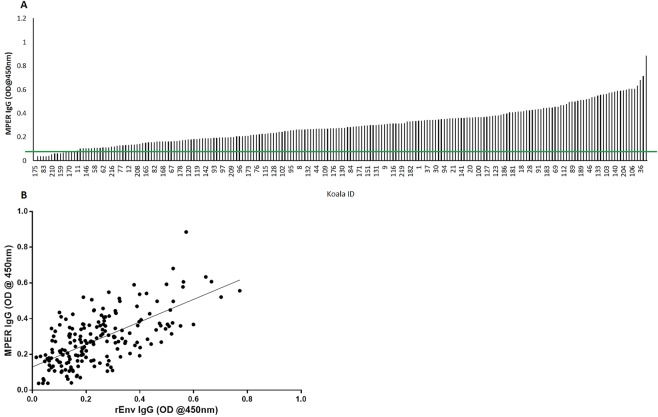
(**A**) MPER IgG levels measured at 450 nm OD in serum samples of koalas (n = 197) harboring KoRV-A. The green line indicates the assay background (OD of 0.12, determined from taking the mean plus two times the standard deviation of three KoRV negative koalas). (**B**) Correlation between the levels of MPER IgG (measured at 450 nm OD) and rEnv IgG (measured at 450 nm OD) in koalas (R^2^ = 0.44, p = 0.0001, n = 197).

While three of the four distinct epitopes recognized in this study (peptides 10–11, 24–25, and 29–30) have been shown to induce neutralizing antibodies in FeLV, PERV and HIV-1, peptides 42–45 present in the MPER have been shown to induce neutralizing antibodies against the above retroviruses, as well as KoRV, in experimental immunization studies in non-koala models^46^. Therefore, we screened for antibodies to this epitope in a larger cohort of our koalas (n = 197). A 31-amino acid synthetic peptide spanning the MPER of KoRV p15E was synthesized and used as an antigen to measure antibody levels directed against this region. We found that the pattern of anti-MPER IgG levels was similar to IgG levels against the full length env protein reported in Figs 2 and 8A. To accurately compare the anti-MPER IgG to the anti-rEnv IgG, we measured the anti-rEnv IgG levels at a serum dilution of 1:1000, similar to the anti-MPER IgG ELISA (Supplementary Fig. 4). We observed a significant correlation between MPER and rEnv IgG levels (R^2^ = 0.437, p < 0.0001, n = 197) (Fig. 8B).

## Discussion

Koala retrovirus (KoRV) poses a significant threat to koala populations. KoRV-A is present in all northern koala populations in Australia, with some koalas in this area co-infected with KoRV-B^16^. All samples included in this study were positive for KoRV-A provirus, while only 27% were positive for KoRV-B provirus. This finding is consistent with infection rates in northern koala populations reported in previous studies^16,47^.

To further understand how the koala’s immune system responds to KoRV infection, we investigated whether koalas with both endogenous and exogenous KoRV could make antibodies against KoRV. A previous study, using a western blot assay and small sample numbers (n = 16), suggested that no antibodies were made against KoRV in northern koalas (harboring both endogenous and exogenous KoRV)^41^. However, a subsequent study using similar methods found that nearly all tested koalas (6 of 7) had detectable antibodies against at least one of the KoRV-A proteins used in the study^48^. The authors explained that they were able to detect anti-KoRV antibodies because they had access to additional reagents and controls. In the current study, we sought to settle this discrepancy by employing a more sensitive and quantitative serological assay (ELISA) and to assess a much larger number of koalas (n = 235). Using our techniques, we found that most koalas do make a detectable antibody response to KoRV. While a limited number of koalas in our cohort (5%) appeared to make little or no anti-KoRV IgG response, the rest (95%) did make an IgG response specific to KoRV. This is an important finding, as it suggests that koalas possessing an endogenous KoRV variant are not tolerant to KoRV but are capable of making an antibody response against KoRV.

In contrast to most endogenous retroviruses that are millions of years old, KoRV-A is a relatively new germline acquisition (under 50,000 years)^49^ and appears to exist as an endogenous virus that retains infectious properties within northern koalas^11^. Therefore, it is not surprising that KoRV-A, in contrast to ancient quiescent endogenous retroviruses, does not induce immunological tolerance. The expression of endogenous Jaagsiekte sheep retrovirus, a Betaretrovirus, during early stages of ontogeny is thought to contribute to tolerance and absence of serological response later in life^50^. It is currently unknown at what stage and in which cells/tissues during ontogenesis endogenous KoRV proteins become expressed. The lack of tolerance suggests that the viral proteins are expressed after the establishment of some immune function or acquisition of passive immunity, possibly via immunoglobulin-rich milk^51^ from the mother (as is the case with opossums, *Monodelphis domestica*)^52^.

A relationship between age and host immunity has been shown in many infection models^33,53^. Typically, dependent juvenile koalas become physically independent of their mothers around one year of age, reach sexual maturity at approximately two years of age and are considered adults at four years. The average life expectancy for koalas in the wild is around 13 years^54^. We investigated possible correlations between antibodies against KoRV and koala age. Analysis showed a gradual increase in antibody levels against KoRV as the animals aged and progressed from dependent juveniles to adults, with antibody levels peaking at around six to eight years of age and then declining gradually as the koalas continued to age further. In addition, when we analyzed a set of koalas over time, a similar antibody pattern to the general population was observed. An age-related reduction in antibodies against HERV-K in women has recently been shown, with the authors suggesting anti-HERV-K serum antibodies can be an important parameter reflecting a person’s age^53^. When we examined specific epitopes over time in our study, we found juvenile animals (aged about one year) and elderly animals (nine years and above) recognized fewer epitopes compared to adult koalas (about four years of age). Since it has been shown that while the total number of B cells is relatively constant for most of life^55^, it is possible that age-related changes in antibody repertoire quality may be responsible for changes in response to natural infection or vaccination^56^. Also, aging has been associated with a decreased diversity, and even loss of high affinity antibodies, upon immunization^57^.

In this study, we found that koalas with detectable levels of KoRV-B had slightly higher anti-KoRV IgG levels compared to koalas in which KoRV-B was not detected. A possible explanation for this observation could be that koalas harboring KoRV-B express more virus (due to the presence of more enhancers in its LTRs) and, therefore, more antigens, than those harboring solely KoRV-A. Another possible explanation is the presence of the CETTG motif in KoRV-B env protein^1^. The CETTG motif is present in all infectious gammaretroviruses and has been shown to be more pathogenic than the CETAG/CGTAG motif present in KoRV-A and other endogenous gammaretroviruses^7^. We hypothesize that the presence of this more pathogenic motif in KoRV-B may lead to the induction of a stronger immune response, and hence the higher levels of anti-KoRV-IgG, seen in KoRV-B positive koalas in this study.

Retroviral infections typically result in the induction of virus-specific antibodies^58^ capable of preventing virus replication^59^. An inverse relationship has been reported to exist between viral load and immune response in some instances^28,30,60^. Similar to these studies in other retroviruses, we observed an inverse relationship between total circulating KoRV load and anti-KoRV IgG levels. However, when we expanded this population-level survey to individual koalas tested over time, the viral load/anti-KoRV IgG relationship was less clear.

Three epitopes recognized across all age groups were identified in this study, one within the fusion peptide proximal region and two within the immunosuppressive domain. Interestingly, these regions have been shown to induce neutralizing antibodies following vaccination in other retroviruses, such as FeLV, HIV-1 and PERV^46,61,62^. This offers great promise for the KoRV antibodies to these epitopes; however further experiments should be set up to determine the actual neutralizing potentials of these epitopes in KoRV. A sequence alignment of the epitopes among different KoRV subtypes shows a high degree of sequence conservation. This is important as it suggests that these epitopes could be useful in designing a multivalent/multi-subtype vaccine against KoRV.

CKS-17, with the amino acid sequence LQNRRGLDLLFLKEGGL, is a highly conserved region on the immunosuppressive domain in many retroviruses. It has been shown to possess the ability to suppress cell-mediated immunity, inhibit interleukin 2, interferon gamma and tumor necrosis factor production, as well as inhibit lymphocyte proliferation and B cell activation^63–66^. This sequence is present in KoRV p15E region epitope two (peptides 24–25) and was recognized by koalas in this study. Given the importance of this region to the host-virus interaction for many retroviruses, the strong koala antibody response to the CKS-17 region is highly encouraging and warrants further investigation.

We recently reported an anti-KoRV vaccine based on the recombinant KoRV env protein capable of inducing neutralizing antibodies and reduction of viral load in koalas with KoRV infection^42^. We were interested in determining if the antibodies produced by koalas harboring endogenous and exogenous KoRV from the present study were different from the antibodies induced by vaccination of koalas believed to be harboring only exogenous KoRV. It has been suggested that optimizing vaccines to elicit a broad range of targets on a pathogen might result in a better protective response^67^. Some epitopes (24–26, 42–45) in the immunosuppressive domain and the MPER region recognized by the vaccinated koalas were also recognized by the non-vaccinated koalas suggesting that natural KoRV infection may induce the same antibodies against KoRV as vaccination. We also found that vaccinated koalas recognized some epitopes (such as epitope 32) that were not recognized by non-vaccinated koalas. It remains to be seen if unique epitopes like this may explain the difference between natural and vaccine-induced response to viral infections.

Critical to developing an efficacious therapeutic vaccine against KoRV is understanding how the koala’s immune system responds to natural KoRV infection. The fact that KoRV is ubiquitous in northern Australia (and may be spreading in southern koala populations)^68^, makes this task even more urgent. This study showed that koalas with both endogenous and exogenous KoRV are not tolerant to KoRV infection, as they are able to make antibodies. We also report an inverse relationship between anti-KoRV IgG and KoRV viral load. These are important findings, as they highlight the possibility of developing a therapeutic vaccine to boost the natural anti-KoRV IgG response that may reduce circulating viral load. The distinct epitopes identified in this study can be used as the basis for the development of a multivalent KoRV vaccine. In conclusion, this study suggests that all koalas with KoRV may benefit from a vaccine designed to boost the natural antibody response in these animals.

## Materials and Methods

### Animals and sampling

Koalas included in this study were from two wild koala populations, geographically 100 kilometres apart, Gold Coast City Region (GCCR) and Moreton Bay (MBR) in south east Queensland. Samples were collected from a total of 235 koalas between 2012 and 2017; 28 from GCCR and 207 from MBR. These koalas were captured, examined by qualified veterinarians, tagged and released back into the wild. Many of these koalas from the MBR population were recaptured at regular intervals, usually every six months, during the 5-year study period. Blood samples were collected during clinical examination and stored at -20 °C. Serum and plasma were obtained from whole blood and stored at -20 °C. A more detailed description of sampling procedure has been previously reported^69^. Also included in this study is a previously described group of KoRV-A positive southern koalas (n = 3) sourced from Adelaide, South Australia, and vaccinated with a tri-adjuvanted recombinant KoRV envelope protein vaccine^42^. These koalas received two doses of the vaccine subcutaneously, four weeks apart. Blood samples for the current study were obtained on day 0 (pre-vaccination) and 8 weeks post-vaccination. All procedures were approved by the relevant authorities (University of the Sunshine Coast’s Animal Ethics Committee: AN/A/13/80 and by the Queensland Government, Scientific Purposes Permit, WISP11532912). All experiments were performed in accordance with relevant guidelines and regulations.

### KoRV testing and viral load quantification

Using genomic DNA extracted from whole blood, all koalas in this study were screened for KoRV infection following our previously described protocol^69^. Briefly, DNA was extracted from 200 µl of whole blood using Qiagen’s QIAamp DNA minikit following manufacturer’s instructions. KoRV subtypes A and B were screened for using a universal forward primer (5′-TCCTGGGAACTGGAAAAGAC-3′) and subtype-specific reverse primers (KoRV-A, 5′-GGGTTCCCCAAGTGATCTG-3′, 321-bp product; KoRV-B, 5′-GGCGCAGACTGTTGAGATTC-3′, 271-bp product). Standard PCR was carried out using the HotStarTaq Plus master mix kit (Qiagen).

Quantification of circulating total KoRV viral RNA using the *pol* gene as the PCR target was carried out by modifying existing protocols from Waugh *et al*.^16^. Briefly, Viral RNA was extracted from plasma stored in RNALater using the Qiagen viral RNA mini kit. Contaminating DNA was removed using TURBO DNA-free (Life technologies, USA). cDNA synthesis of RNA prepared from plasma was conducted using iScript^TM^ cDNA synthesis kit (Bio-Rad, USA). Viral RNA load was thereafter quantified using Tarlinton *et al*.^4^ pol gene primers (forward: 5′- 5′-TTGGAGGAGGAATACCGATTACAC-3′ and reverse 5′-GCCAGTCCCATACCTGCCTT-3′) using iTaq™ Universal SYBR® Green Supermix (Bio-Rad, USA). Standards of known concentration (10^8^ to 10^1^ copy numbers) of purified KoRV pol gene PCR product were prepared for each run and the results were normalized against the standard curve. PCR reactions were carried out on a CFX 96 Touch System (Bio-Rad, Australia) with the following cycling conditions: initial denaturation of 95 °C for 5 min, and then 40 cycles of denaturing at 94 °C for 15 s, annealing at 60 °C for 30 s, and extension at 72 °C for 30 s. All procedures were carried out following the manufacturer’s instructions.

### Anti-KoRV IgG ELISA

Two previously described ELISAs specific to anti-KoRV IgG^42^ were used in this study. The first ELISA was used for measuring anti-KoRV IgG in koala serum using a recombinant KoRV envelope (rEnv) protein (Fig. 1) as antigen. Briefly, serum antibodies that may be directed against the glutathione S-transferase (GST) component of the rEnv were initially depleted by incubating sera with GST at 4 °C overnight. The serum samples were then serially diluted two-fold on a separate plate, starting from an initial dilution of 1:200, and added to the test wells containing rEnv protein. Sheep anti-koala IgG and donkey anti-sheep IgG were thereafter added followed by the addition of TMB substrate for colour development. The reaction was subsequently stopped using 1 M H_2_SO_4_ and colour development measured at optical density of 450 nm. To make the end point titre measurement as precise as possible, the optical densities from the serial dilutions of each sample were plotted on a graph and used to define a best fit line. The point where that best fit line crossed the threshold of positivity (optical density of the no sample controls) was interpolated from the line and reported as the end point titre. This was calculated with GraphPad Prism 6 (GraphPad Software, Inc, La Jolla, CA).

A second ELISA measured koala IgG specific to a 31-amino acid (LKERLDKRQL EHQKNLSWYE GWFNRSPWLT T) synthetic peptide of KoRV that spans the membrane proximal external region (MPER) of the KoRV env protein. For this assay we report the optical density for a single dilution (1:1000) of the test serum samples minus the optical density of the blank wells (no sample controls). In order to accurately compare the anti-MPER IgG to the anti-rEnv IgG, we developed a single dilution variant of the anti-rEnv IgG ELISA (1:1000 of test sera), similar to the anti-MPER IgG ELISA. This extra assay was set up to ensure the antibody levels against the different test antigens used for comparison were obtained using similar methodology. Assay background was determined for all assays by calculating the mean plus two times the standard deviation of serum IgG titers of three KoRV negative koalas. The KoRV negative koalas were sourced from Adelaide, South Australia and were screened and confirmed negative for KoRV using a genomic DNA conventional PCR assay targeting the pol gene, and quantitative PCR of viral RNA targeting the KoRV-A and KoRV-B env gene as per^42^.

### Epitope mapping

Forty-eight overlapping 15-mer peptides with three amino acids offset were made using the amino acid sequence representing the ectodomain of p15E subunit of KoRV env protein (Fig. 1c). The peptide library was constructed by Mimotopes (Melbourne, Australia) as biotinylated peptides which included the hydrophilic tetrapeptide SGSG between the biotin and sequence of interest and added to streptavidin-coated plates at a concentration of 2 µg/well^42^. The plates were incubated for two hours at room temperature and thereafter washed four times with phosphate buffered saline (PBS) containing 0.1% Tween-20 (PBST). Sera was added to the plates at 1:1000 and incubated overnight at 4 °C. The plates were thereafter washed four times with PBST. Sheep anti-Koala IgG^70^ was added at a dilution of 1:8000 and followed by a one-hour incubation at room temperature and four washes with PBST. Secondary antibody, donkey anti-sheep HRP IgG (*In Vitro* Technologies, Australia), was added at a dilution of 1:5000 in PBS, incubated for 1 hour at room temperature and subsequently washed four times with PBST and two times with PBS. Fifty µl of Tetramethylbenzidine Liquid Substrate (Sigma-Aldrich) was added to each well and the reaction was stopped after 10 minutes using 50 μl of 1 M H_2_SO_4_. Optical density was read at 450 nm (EnSpire Multimode Plate Reader, PerkinElmer, Australia) and titres thereafter calculated (GraphPad Prism 6).

## Supplementary information

Supplementary Information

## Data Availability

The data that support the findings of this study are available from the authors on reasonable request.
