# Supplementary material for: Antibody response against koala retrovirus (KoRV) in koalas harboring KoRV-A in the presence or absence of KoRV-B
Source: Sci Rep. 2019 Aug 27;9:12416. doi: 10.1038/s41598-019-48880-0 (PMC6711960; doi:10.1038/s41598-019-48880-0)

Supplementary material for:

Antibody response against koala retrovirus (KoRV) in koalas harboring KoRV-A in the presence or absence of KoRV-B

O Olagoke[1], BL Quigley[1], MV Eiden[2], P Timms[1]*

[1]Genecology Research Center, University of the Sunshine Coast, 90 Sippy Downs Drive, Sippy Downs, 4556, Queensland, Australia. [2] Section on Directed Gene Transfer, Laboratory of Cellular and Molecular Regulation, National Institute of Mental Health, National Institutes of Health, Bethesda, Maryland, USA, *Corresponding author: ptimms@usc.edu.au

Supplementary Figure 1: Anti-KoRV IgG levels expressed as end-point titers in koala serum samples from two geographical locations.

Supplementary Figure 2: Anti-KoRV IgG levels expressed as end-point titers in serum samples from individual koala (n = 14).

Supplementary Figure 3: KoRV viral RNA load and anti-KoRV serum IgG titers over time in 15 koalas

Supplementary Figure 4: Anti-KoRV rEnv IgG levels (OD @ 450nm) in serum samples of KoRV-A positive koalas (n = 197).

Supplementary Figure 1: Anti-KoRV IgG levels expressed as end-point titers in koala serum samples from two geographical locations; Gold Coast City Council (GCC) (n = 28) and Moreton Bay region (MBR) (n = 207). Average serum IgG titers were compared and presented as mean ± SD. The level of significance was measured as $p = 0.287$ using unpaired student t-test.

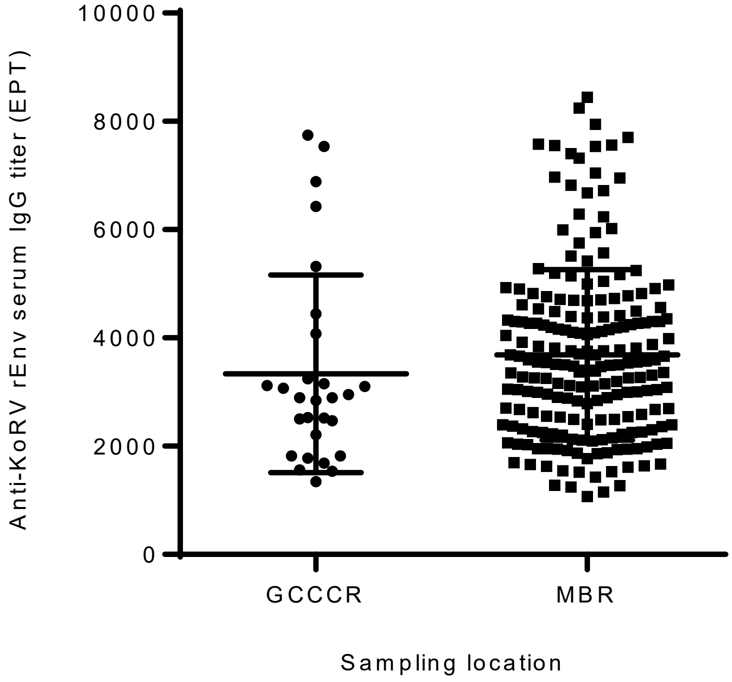

Supplementary Figure 2: Anti-KoRV IgG levels expressed as end-point titers in serum samples from individual koala (n = 14). Serum IgG titer was measured over a minimum of three years in each koala.

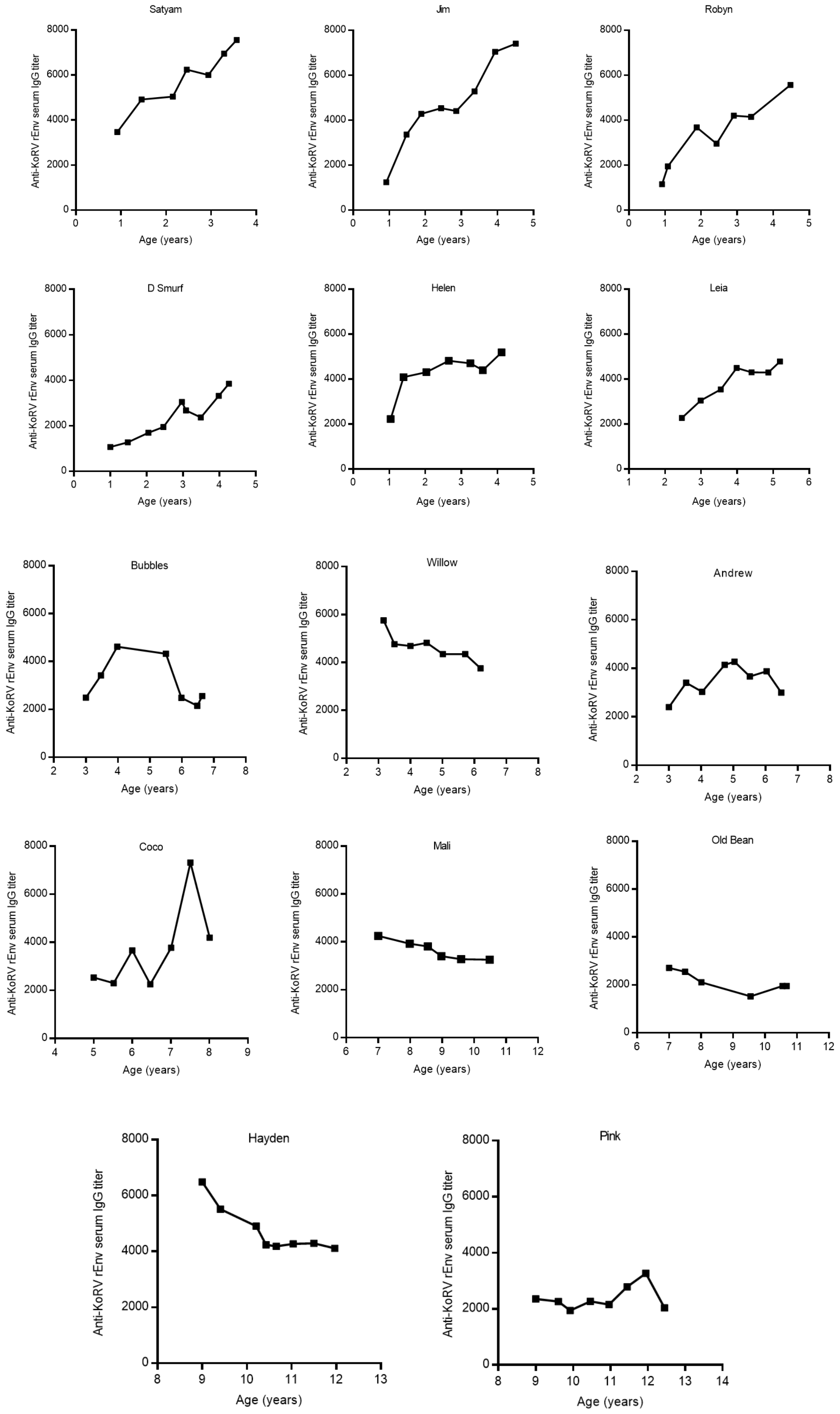

Supplementary Figure 3: KoRV viral RNA load and anti-KoRV serum IgG titers over time in 15 koalas

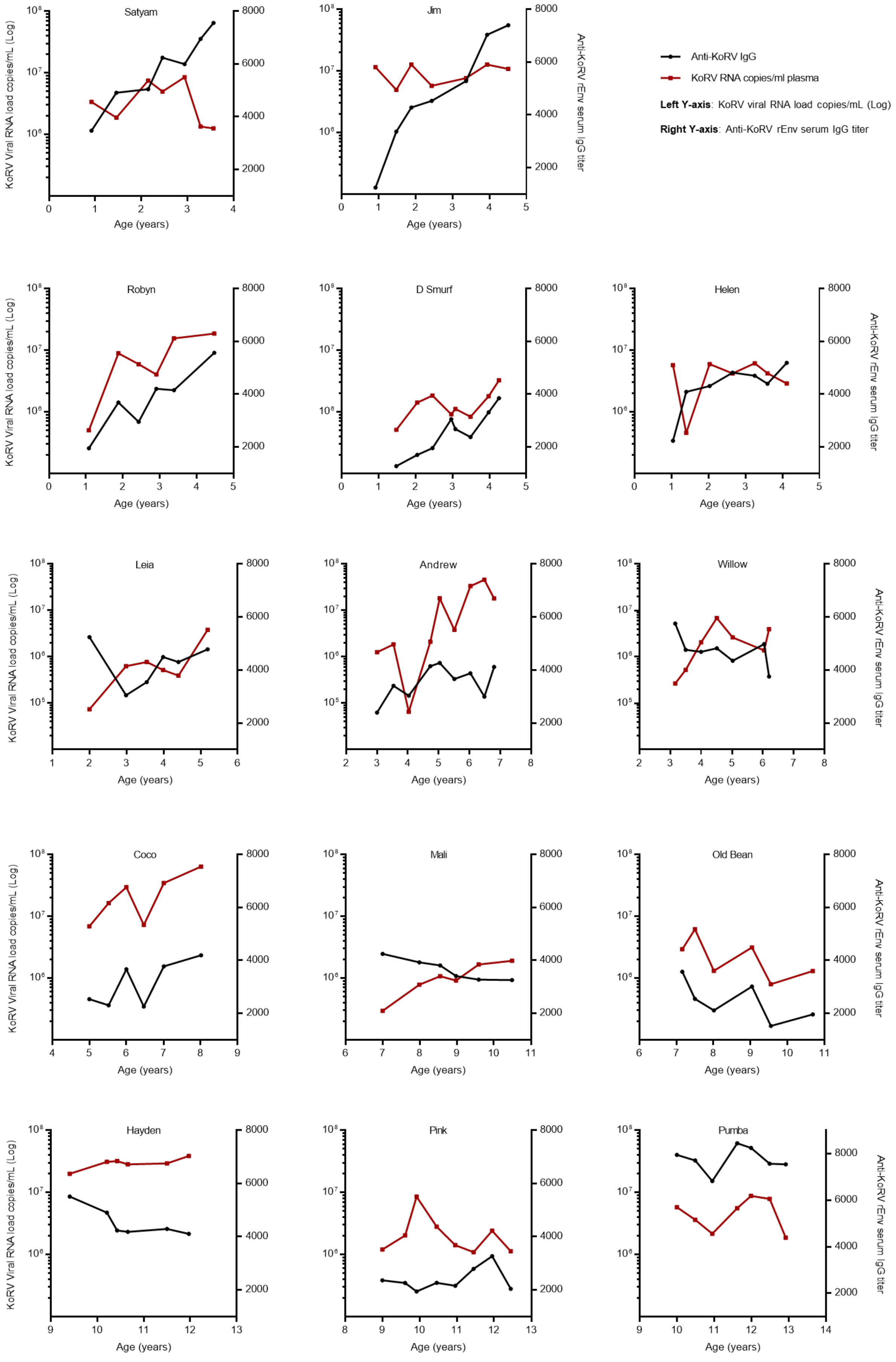

Supplementary Figure 4: Anti-KoRV rEnv IgG levels (OD @ 450nm) in serum samples of KoRV-A positive koalas (n = 197). The green line indicates the assay background

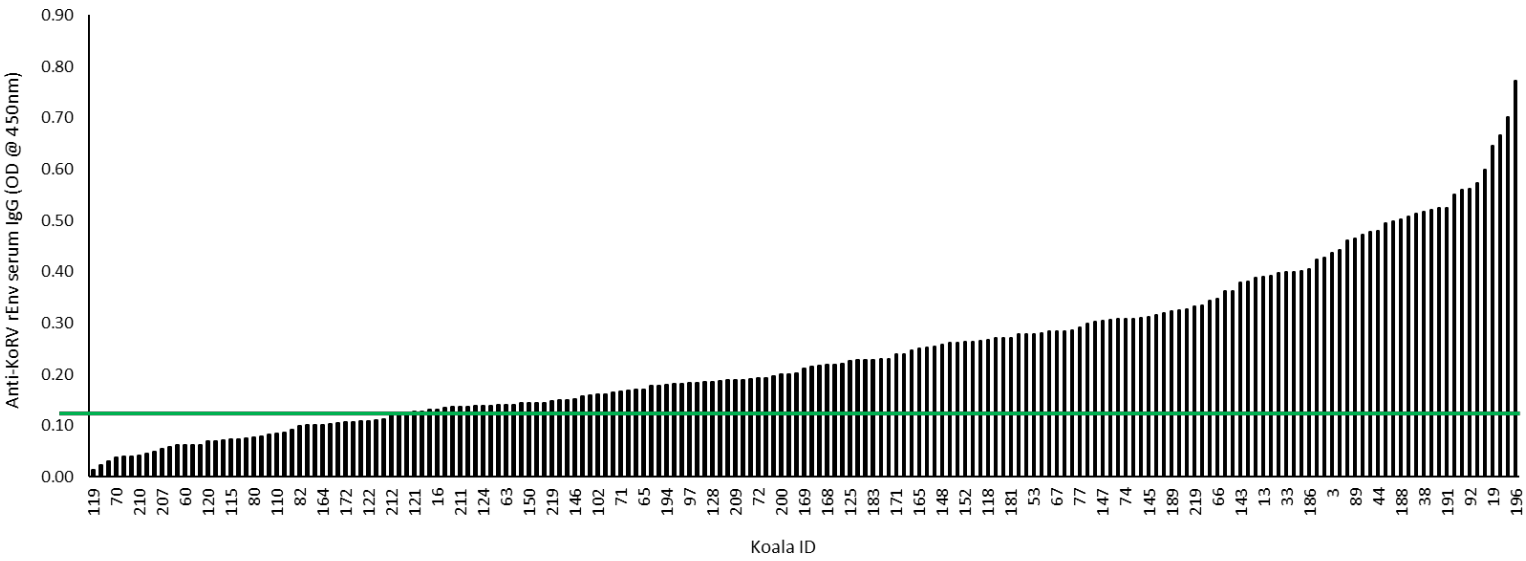

Supplement: Supplementary file 1 — Supplementary Information [file 41598_2019_48880_MOESM1_ESM.pdf]
